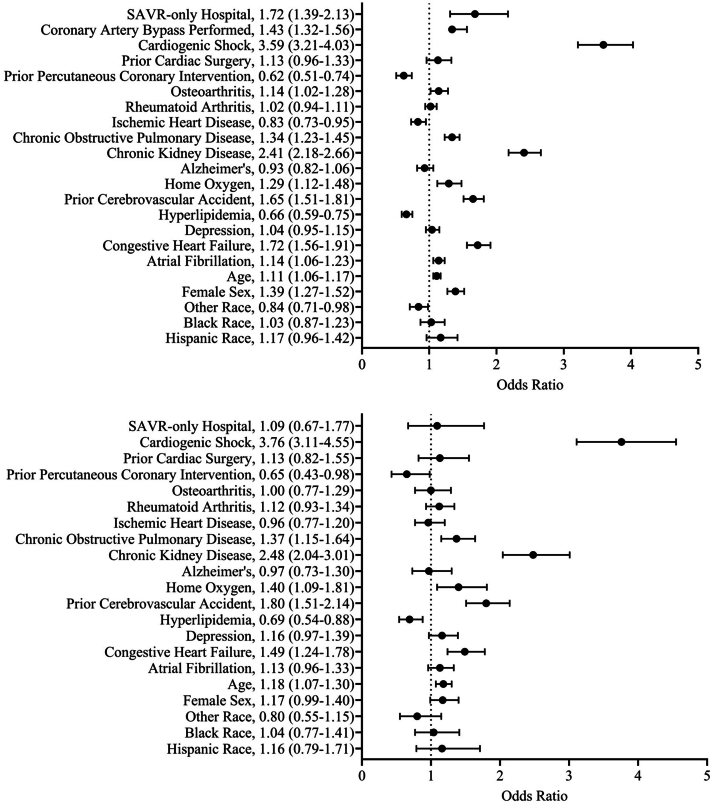# Surgical Aortic Valve Outcomes With Transcatheter Aortic Valve Replacement Hospital Status

**DOI:** 10.1016/j.atssr.2025.07.002

**Published:** 2025-07-30

**Authors:** Maxwell C. Braasch, Fengxian Wang, R.J. Waken, Karen E. Joynt Maddox, Alexander A. Brescia, Tsuyoshi Kaneko

**Affiliations:** 1 Division of Cardiothoracic Surgery, Department of Surgery, Washington University in St Louis, St Louis, Missouri; 2 Center for Advancing Health Services, Policy, and Economics Research, Washington University in St Louis, St Louis, Missouri; 3 Division of Biostatistics, Washington University in St Louis, St Louis, Missouri

## Abstract

**Background:**

Both surgical aortic valve replacement (SAVR) and transcatheter aortic valve replacement (TAVR) are treatments of aortic stenosis. There remain hospitals that perform SAVR and not TAVR. The study objective was to compare SAVR outcomes at SAVR-only hospitals with those at SAVR/TAVR hospitals.

**Methods:**

Medicare beneficiaries who underwent SAVR from January 2018 to June 2023 were analyzed. Logistic regression analyses were performed to determine whether 30-day and 1-year mortality of patients undergoing SAVR differed between SAVR-only and SAVR/TAVR hospitals from 2021 to 2023.

**Results:**

A total of 98,003 SAVRs occurred from 2018 to 2023, including 94,170 performed at SAVR/TAVR hospitals. From 2021 to 2023, 30-day and 1-year mortality after SAVR was higher at SAVR-only hospitals than at SAVR/TAVR hospitals (7.3% vs 5% [*P* < .0001]; 14.1% vs 8.8% [*P* < .0001]). All SAVRs at SAVR-only hospitals had higher odds of both 30-day (odds ratio [OR], 1.68 [CI, 1.31-2.17]) and 1-year (OR, 1.77 [CI, 1.44-2.19]) mortality compared with SAVRs performed at SAVR/TAVR hospitals, whereas there was no difference in 30-day (OR, 1.22 [CI, 0.71-2.10]) or 1-year mortality (OR, 0.82 [CI, 0.58-1.19]) for isolated SAVRs.

**Conclusions:**

Mortality after all SAVRs is higher at SAVR-only hospitals than at SAVR/TAVR hospitals, but not for isolated SAVR. Further research is needed to understand whether the Center of Excellence concept is the best avenue to improve SAVR mortality.

## Visual Abstract

**Figure:**
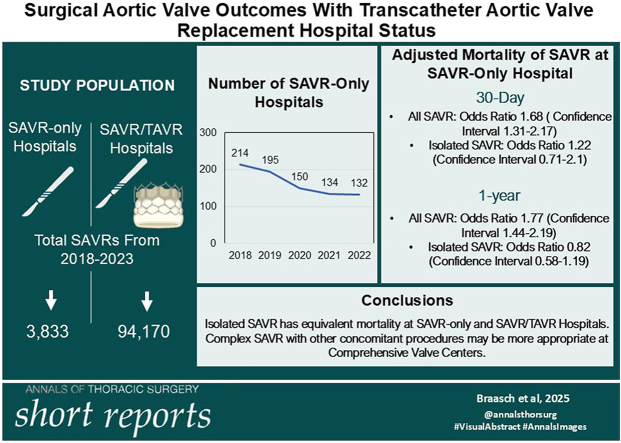

**In Short**

▪ Postoperative mortality is higher after all surgical aortic valve replacement (SAVR) procedures at SAVR-only hospitals than at SAVR/transcatheter aortic valve replacement hospitals, but not for isolated SAVR.
▪ Further research is needed to understand whether the Center of Excellence concept is the best avenue to lower mortality after SAVR.

Transcatheter aortic valve replacement (TAVR) is an alternative to surgical aortic valve replacement (SAVR), performed more frequently than SAVR in the United States.^1^ The Centers for Medicare & Medicaid Services (CMS) implemented national coverage determinations (NCDs) for TAVR,^2^ dictating reimbursement for TAVR. The American College of Cardiology and The Society of Thoracic Surgeons introduced guidelines on creating sustainable and effective TAVR practices, including the concept of the multidisciplinary heart team.^3^

Critical analysis to determine the appropriate aortic intervention for patients is essential. Given the recent expansion of TAVR to low-risk patients^4^ and changes to NCDs in 2019,^2^ improved understanding of SAVR at SAVR-only hospitals in a contemporary sample is needed. The objective of this study was to evaluate outcomes of both isolated SAVR and all SAVRs at SAVR-only hospitals compared with SAVR/TAVR hospitals.

## Material and Methods

A retrospective design was implemented. The Washington University in St. Louis Institutional Review Board determined this study to be exempt and informed consent was waived (IRB #202301098; approved January 19, 2023). The Virtual Research Data Center was used to access Medicare inpatient claims for fee-for-service beneficiaries with *International Classification of Diseases, Tenth Revision* procedure codes indicating a SAVR procedure between January 2018 and June 2023, and American Hospital Association survey data were used to describe hospital characteristics (Supplemental Table 1). Comorbidities were ascertained by diagnosis codes included in the 27 chronic conditions segment in a 2-year lookback period in CMS claims not including the SAVR hospitalization. Two study groups were created: SAVR-only hospitals and SAVR/TAVR hospitals. If a hospital performed at least 1 TAVR during a 1-year period through TAVR procedure code query, it was considered a SAVR/TAVR hospital for that year. If not, it was considered a SAVR-only hospital.

Postprocedural SAVR mortality through multivariable modeling at 30 days, 90 days, and 1 year was analyzed for SAVR procedures occurring between January 2021 and June 2023 to reflect the changing landscape of aortic valve replacement procedures. For SAVR performed at SAVR/TAVR hospitals, postprocedural SAVR mortality at 30 days and 1 year was compared before and after the first TAVR performed at each hospital. January 2020 was chosen instead of January 2021 to allow analysis of 1-year outcomes for more SAVRs. Logistic regression models using the generalized estimating equations describing mortality for all SAVR claims based on whether the procedure occurred in a SAVR-only hospital and beneficiary characteristics while clustering by hospital to account for within-hospital similarities were performed.

## Results

**Figure 1:**
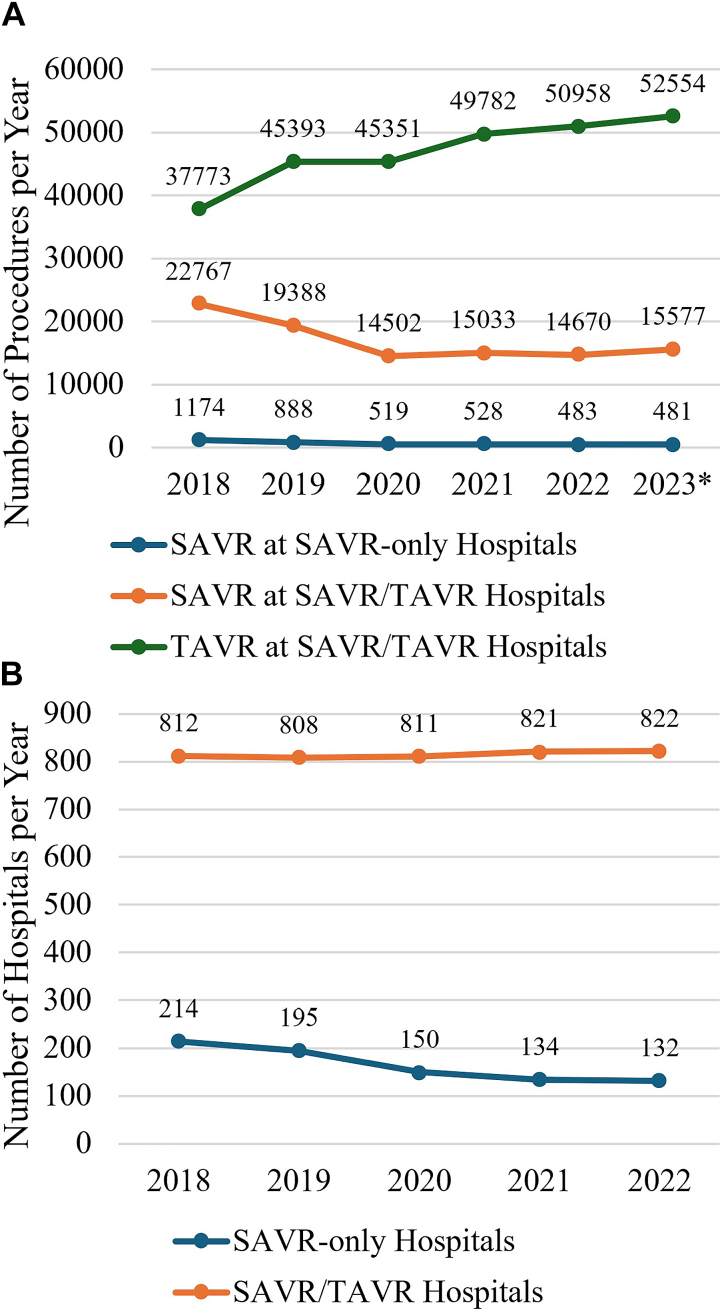
(A) Number of surgical aortic valve replacements (SAVRs) and transcatheter aortic valve replacements (TAVRs) performed at SAVR-only and SAVR/TAVR hospitals and (B) number of SAVR-only and SAVR/TAVR hospitals. ∗2023 is annualized on the basis of 6 months of data.

A total of 98,003 SAVRs occurred from 2018 to 2023 (Figure 1). Each year, most SAVRs were performed at SAVR/TAVR hospitals. The total number of hospitals in the SAVR-only group decreased every year while the number of hospitals in the SAVR/TAVR group stayed relatively constant.

Patients who underwent SAVR at SAVR-only hospitals had a higher preoperative comorbid disease burden of hypertension, diabetes mellitus, chronic obstructive pulmonary disease, ischemic heart disease, home oxygen use, arthritis, Alzheimer’s disease, and depression (all *P* < .05; Table). A higher percentage of patients who underwent SAVR at SAVR-only hospitals underwent concomitant coronary artery bypass graft, but fewer underwent concomitant valvular or aortic operations (all *P* < .05) compared with those at SAVR/TAVR hospitals.

**Table:** Patient, Hospital, and Outcome Data of SAVR at SAVR-Only Hospitals and SAVR/TAVR Hospitals From 2018 to 2023

Variable	SAVR at SAVR-Only Hospital (n = 3833)	SAVR at SAVR/TAVR Hospital (n = 94,170)	*P* Value
Age, y			.13
<65	339 (8.8)	8608 (9.1)	
65-74	2156 (56)	54,185 (58)	
75-84	1236 (32)	29,247 (31)	
≥85	102 (2.7)	2130 (2.3)	
Male sex	2599 (68)	64,453 (68)	.40
Race			<.0001
Non-Hispanic White	3187 (83)	81,215 (86)	
Black/African American	206 (5)	4215 (4.5)	
Hispanic	258 (6.7)	3756 (4)	
Asian/Pacific Islander	53 (1.4)	1397 (1.5)	
American Indian/Alaska Native	31 (0.8)	349 (0.37)	
Unknown	77 (2)	2619 (2.8)	
Other	21 (0.55)	619 (0.66)	
Preoperative comorbidities			
Hyperlipidemia	3345 (87)	83,425 (89)	.012
Hypertension	3604 (94)	87,537 (93)	.011
Diabetes mellitus	1825 (48)	39,543 (42)	<.0001
Prior stroke	594 (16)	15,173 (16)	.31
Chronic obstructive pulmonary disease	1359 (35)	28,138 (30)	<.0001
Chronic kidney disease	2115 (55)	51,994 (55)	.97
Atrial fibrillation	1415 (37)	38,143 (41)	<.0001
Ischemic heart disease	3401 (89)	81,689 (87)	.0004
Heart failure	2106 (55)	55,738 (59)	<.0001
Home oxygen	288 (7.5)	5610 (6)	<.0001
Rheumatoid arthritis/osteoarthritis	2535 (66)	59,274 (63)	<.0001
Alzheimer’s disease	321 (8.4)	6966 (7.4)	.024
Osteoporosis	392 (10)	9217 (9.8)	.37
Depression	899 (23)	20,231 (21)	.004
Cardiogenic shock at hospital presentation	225 (5.9)	8787 (9.3)	<.0001
Prior percutaneous coronary intervention	319 (8.3)	7474 (7.9)	.39
Prior cardiac surgery	211 (5.5)	5847 (6.2)	.076
Dually enrolled in Medicaid (proxy for individual poverty)	521 (14)	9941 (11)	<.0001
Rurality			<.0001
Metropolitan (≥50,000 urban cluster)	3580 (93)	92,230 (98)	
Micropolitan (10,000-50,000 cluster)	238 (6.2)	1849 (2)	
Rural (cluster ≤9999)	15 (0.4)	91 (0.1)	
Bed size of hospital			<.0001
<100	285 (7.4)	2262 (2.4)	
100-399	3382 (88)	29,956 (32)	
≥400	166 (4.3)	61,952 (66)	
Emergent or urgent hospitalization	1110 (29)	24,031 (25)	<.0001
Concomitant coronary artery bypass graft	1917 (50)	38,750 (41)	<.0001
Concomitant nonaortic valve procedure	246 (6.4)	9654 (10)	<.0001
Concomitant aortic operation	280 (7.3)	13,563 (14)	<.0001
Postoperative mortality			
30-day mortality	279 (7.3)	4676 (5)	<.0001
1-year mortality	143 (14.1)	2621 (8.8)	<.0001

Values are reported as number (%).

SAVR, surgical aortic valve replacement; TAVR, transcatheter aortic valve replacement.

**Figure 2:**
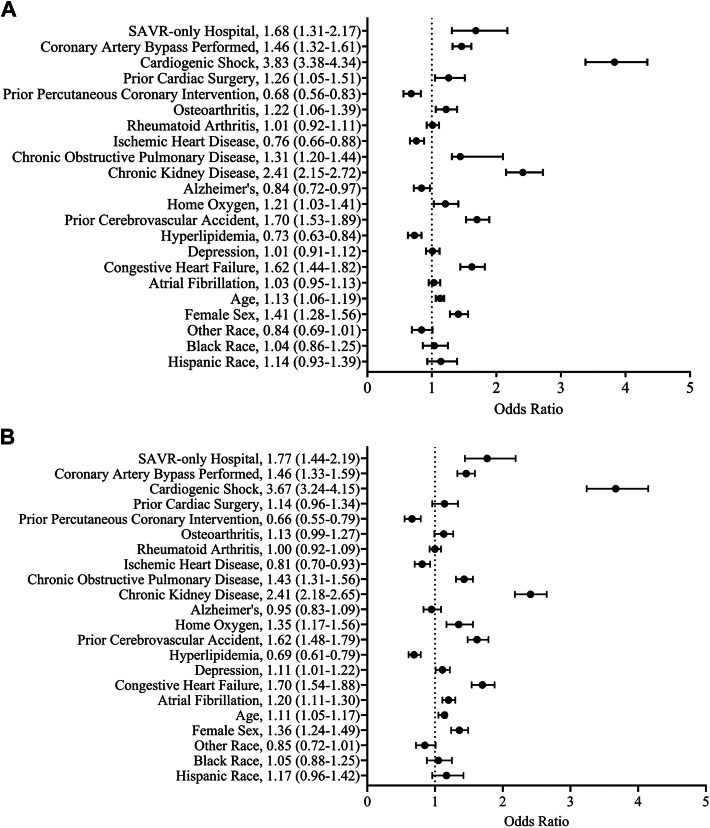
Multivariable analysis of mortality after all surgical aortic valve replacement (SAVR) procedures at (A) 30 days and (B) 1 year.

**Figure 3:**
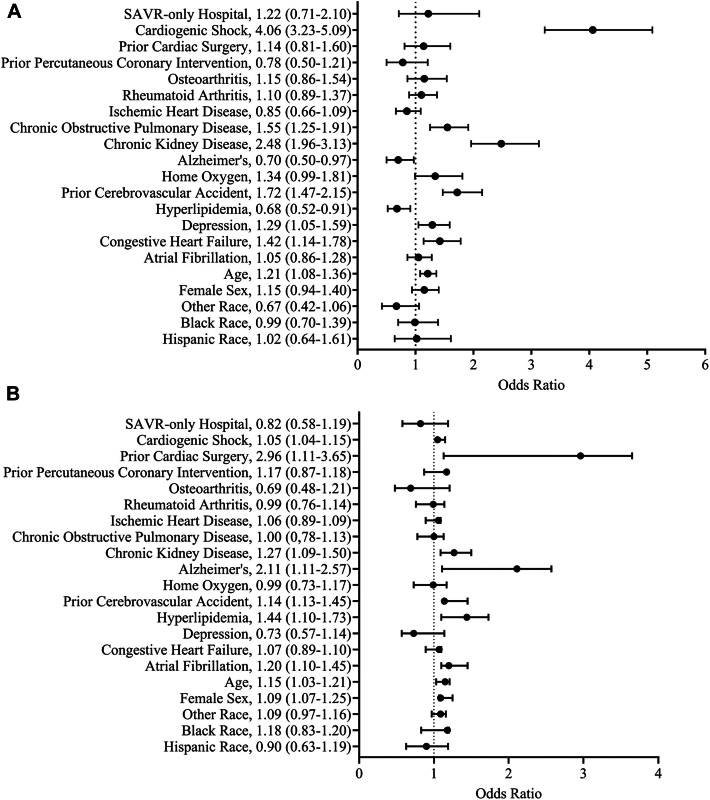
Multivariable analysis of mortality after isolated surgical aortic valve replacement (SAVR) at (A) 30 days and (B) 1 year.

Raw mortality after all SAVRs at SAVR-only hospitals was higher at 30 days (7.3% vs 5%; *P* < .001) and 1 year (14.1% vs 8.8%; *P* < .001) compared with SAVR/TAVR hospitals (Table). Raw mortality after isolated SAVR was similar at SAVR-only hospitals at 30 days (3.6% vs 3.2%; *P* = .76) and 1 year (5.9% vs 5.7%; *P* = .99) to SAVR/TAVR hospitals (Supplemental Table 2). Raw 30-day mortality at SAVR/TAVR hospitals after all SAVRs was not different after the first TAVR performed (6.2% vs 6.2%; *P* > .99), but 1-year mortality was lower after the first TAVR was performed (7.6% vs 11.6%; *P* = .047; Supplemental Table 3). SAVRs occurring at SAVR-only hospitals were associated with higher odds of mortality at 30 days (odds ratio [OR], 1.68 [95% CI, 1.31-2.17]) and 1 year (OR, 1.77 [95% CI, 1.44-2.19]) compared with SAVRs performed at SAVR/TAVR hospitals (Figure 2). Isolated SAVR at SAVR-only hospitals was not associated with higher odds of mortality at 30 days (OR, 1.22 [95% CI, 0.71-2.1]) or 1 year (OR, 0.82 [CI, 0.58-1.19]) than at SAVR/TAVR hospitals (Figure 3). Respiratory insufficiency and pacemaker placement were higher after SAVR at SAVR/TAVR hospitals compared with SAVR-only hospitals (both *P* < .05; Supplemental Table 4). Supplemental analysis demonstrated that all SAVRs occurring at SAVR-only hospitals were associated with higher odds of 90-day mortality (OR, 1.72 [95% CI, 1.39-2.13]) than SAVRs performed at SAVR/TAVR hospitals, but isolated SAVRs occurring at SAVR-only hospitals were not associated with higher odds of 90-day mortality (OR, 1.09 [95% CI, 0.67-1.77]) than isolated SAVRs occurring at SAVR/TAVR hospitals (Supplemental Figure).

## Comment

This study demonstrated several important findings. First, most SAVRs were performed at SAVR/TAVR hospitals as opposed to SAVR-only hospitals from 2018 to 2023. Second, multivariable analysis found that all SAVRs at SAVR/TAVR hospitals have higher odds of 30-day, 90-day, and 1-year mortality compared with SAVR performed at SAVR/TAVR hospitals. However, this difference was not observed for isolated SAVRs. These findings are significant as they represent the only comparison of contemporary SAVR outcomes at SAVR-only hospitals compared with SAVR/TAVR hospitals after approval of TAVR for all patient risk groups, highlighting the need to develop strategies to improve outcomes after SAVR at SAVR-only hospitals.

The number of SAVR-only hospitals decreased steadily from 2018 to 2023 while the number of SAVR/TAVR hospitals remained similar, suggesting a plateau of TAVR program establishment with increased TAVR program case volume, given the increase in number of TAVRs. This finding is of particular interest given the recent NCD coverage changes in 2019 that decreased case volume requirements for the establishment of new TAVR centers.^2^

The isolated SAVR 30-day mortality rates of 3.6% in SAVR-only hospitals and 3.2% in SAVR/TAVR hospitals in this study are slightly higher than nationwide data for isolated SAVR at 2.3%^5^^,^^6^; however, these results contain only CMS beneficiaries compared with The Society of Thoracic Surgeons registry cohorts. It is established that higher volume SAVR hospitals have improved post-SAVR mortality.^5^ Although not listed as a separate variable in regression models, the inclusion of the SAVR-only hospital status variable does account for hospital volume in this analysis as these hospitals perform fewer SAVRs. Hospital bed size was also assessed to be highly colinear with SAVR-only hospital status.

There are potential explanations for why all SAVRs at SAVR-only hospitals had worse outcomes compared with those performed at SAVR/TAVR hospitals. First, many of these SAVR-only hospitals are more rural, are smaller, and perform a smaller volume of SAVRs per year. Second, the presence of the heart team required to establish a TAVR center at the SAVR/TAVR centers may provide benefits to the clinical care of patients undergoing SAVR at the same hospitals, both during hospitalization and in follow-up care. Third, patients at higher risk for SAVR at SAVR/TAVR hospitals will have an option to undergo TAVR, which is not an option at SAVR-only hospitals. However, SAVR-only hospitals should be commended for maintaining excellent isolated-SAVR outcomes similar to SAVR/TAVR hospitals despite performing a higher percentage of urgent/emergent SAVRs on a sick cohort of patients.

These findings are not meant to discourage SAVR at SAVR-only hospitals or to indicate individual provider-level deficiencies. In appropriately selected patients, SAVR can be and should continue to be performed safely at SAVR-only hospitals. This is supported by the finding of no difference in mortality for isolated SAVRs performed at SAVR-only and SAVR/TAVR hospitals. Instead, they are meant to engender a discussion about continued development of a national system of comprehensive care for valvular heart disease to improve management of aortic valve disease.^7^ A possible solution is to further commit to performing higher risk SAVRs at comprehensive valve centers per the Center of Excellence concept.^3^ With more high-risk SAVRs performed at comprehensive valve centers, their expertise, experience, and resources may contribute to lower SAVR mortality. This study does not comprehensively evaluate this possible solution but rather helps generate hypotheses to address this issue.

### Limitations

There are limitations that must be considered. This is a retrospective study of CMS data and there are inherent limitations to claims data, including a relative lack of clinical granularity that makes it impossible to replicate risk scores, such as The Society of Thoracic Surgeons predicted risk of mortality scores. In addition, only Medicare patients are included in this study, which produced an older population with a greater comorbidity burden and noticeably different group sizes. Selection bias exists as patients who present to SAVR-only hospitals have no option for TAVR; therefore, there may be bias in outcomes purely based on TAVR availability and not on the characteristics of SAVR. Last, this analysis is unable to quantify how may patients were initially evaluated at a SAVR-only hospital but further managed at a SAVR/TAVR hospital or vice versa.

### Conclusion

Mortality after SAVR at SAVR-only hospitals is higher than at SAVR/TAVR hospitals, but not for isolated SAVR. Further research is needed to understand whether the Center of Excellence concept is the best avenue to further reduce SAVR mortality.

## Figures and Tables

**Supplementary Figure 1:**