# Supplementary material for: Surgical Aortic Valve Outcomes With Transcatheter Aortic Valve Replacement Hospital Status
Source: Ann Thorac Surg Short Rep. 2025 Jul 30;4(1):87–93. doi: 10.1016/j.atssr.2025.07.002 (PMC13100802; doi:10.1016/j.atssr.2025.07.002)
Supplement: Supplementary Table 1 [file mmc1.docx]

Supplemental Table 1. International Classification of Diseases-10 Codes for Utilized for Data Collection

| **Variable** | **International Classification of Disease-10 Codes** |
| --- | --- |
| **SAVR** | 02RF07Z, 02RF08Z, 02RF0JZ and 02RF0KZ |
| **TAVR** | 02RF37Z, 02RF38Z, 02RF3JZ, 02RF3KZ, 02RF37H, 02RF38H, 02RF3JH, 02RF3KH |
| **Hyperlipidemia** | E78.0, E78.00, E78.01, E78.1, E78.2, E78.3, E78.4, E78.41, E78.49, E78.5 (any DX on the claim) |
| **Hypertension** | H35.031, H35.032, H35.033, H35.039, I10, I11.0, I11.9, I12.0, I12.9, I13.0, I13.10, I13.11, I13.2, I15.0, I15.1, I15.2, I15.8, I15.9, I67.4, N26.2 (any DX on the claim) |
| **Diabetes Mellitus** | E08.00, E08.01, E08.10, E08.11, E08.21, E08.22, E08.29, E08.311, E08.319, E08.321, E08.3211, E08.3212, E08.3213, E08.3219, E08.329, E08.3291, E08.3292, E08.3293, E08.3299, E08.331, E08.3311, E08.3312, E08.3313, E08.3319, E08.339, E08.3391, E08.3392, E08.3393, E08.3399, E08.341, E08.3411, E08.3412, E08.3413, E08.3419, E08.349, E08.3491, E08.3492, E08.3493, E08.3499, E08.351, E08.3511, E08.3512, E08.3513, E08.3519, E08.3521, E08.3522, E08.3523, E08.3529, E08.3531, E08.3532, E08.3533, E08.3539, E08.3541, E08.3542, E08.3543, E08.3549, E08.3551, E08.3552, E08.3553, E08.3559, E08.359, E08.3591, E08.3592, E08.3593, E08.3599, E08.36, E08.37X1, E08.37X2, E08.37X3, E08.37X9, E08.39, E08.40, E08.41, E08.42, E08.43, E08.44, E08.49, E08.51, E08.52, E08.59, E08.610, E08.618, E08.620, E08.621, E08.622, E08.628, E08.630, E08.638, E08.641, E08.649, E08.65, E08.69, E08.8, E08.9, E09.00, E09.01, E09.10, E09.11, E09.21, E09.22, E09.29, E09.311, E09.319, E09.321, E09.3211, E09.3212, E09.3213, E09.3219, E09.329, E09.3291, E09.3292, E09.3293, E09.3299, E09.331, E09.3311, E09.3312, E09.3313, E09.3319, E09.339, E09.3391, E09.3392, E09.3393, E09.3399, E09.341, E09.3411, E09.3412, E09.3413, E09.3419, E09.349, E09.3491, E09.3492, E09.3493, E09.3499, E09.351, E09.3511, E09.3512, E09.3513, E09.3519, E09.3521, E09.3522, E09.3523, E09.3529, E09.3531, E09.3532, E09.3533, E09.3539, E09.3541, E09.3542, E09.3543, E09.3549, E09.3551, E09.3552, E09.3553, E09.3559, E09.359, E09.3591, E09.3592, E09.3593, E09.3599, E09.36, E09.37X1, E09.37X2, E09.37X3, E09.37X9, E09.39, E09.40, E09.41, E09.42, E09.43, E09.44, E09.49, E09.51, E09.52, E09.59, E09.610, E09.618, E09.620, E09.621, E09.622, E09.628, E09.630, E09.638, E09.641, E09.649, E09.65, E09.69, E09.8, E09.9, E10.10, E10.11, E10.21, E10.22, E10.29, E10.311, E10.319, E10.321, E10.3211, E10.3212, E10.3213, E10.3219, E10.329, E10.3291, E10.3292, E10.3293, E10.3299, E10.331, E10.3311, E10.3312, E10.3313, E10.3319, E10.339, E10.3391, E10.3392, E10.3393, E10.3399, E10.341, E10.3411, E10.3412, E10.3413, E10.3419, E10.349, E10.3491, E10.3492, E10.3493, E10.3499, E10.351, E10.3511, E10.3512, E10.3513, E10.3519, E10.3521, E10.3522, E10.3523, E10.3529, E10.3531, E10.3532, E10.3533, E10.3539, E10.3541, E10.3542, E10.3543, E10.3549, E10.3551, E10.3552, E10.3553, E10.3559, E10.359, E10.3591, E10.3592, E10.3593, E10.3599, E10.36, E10.37X1, E10.37X2, E10.37X3, E10.37X9, E10.39, E10.40, E10.41, E10.42, E10.43, E10.44, E10.49, E10.51, E10.52, E10.59, E10.610, E10.618, E10.620, E10.621, E10.622, E10.628, E10.630, E10.638, E10.641, E10.649, E10.65, E10.69, E10.8, E10.9, E11.00, E11.01, E11.10, E11.11, E11.21, E11.22, E11.29, E11.311, E11.319, E11.321, E11.3211, E11.3212, E11.3213, E11.3219, E11.329, E11.3291, E11.3292, E11.3293, E11.3299, E11.331, E11.3311, E11.3312, E11.3313, E11.3319, E11.339, E11.3391, E11.3392, E11.3393, E11.3399, E11.341, E11.3411, E11.3412, E11.3413, E11.3419, E11.349, E11.3491, E11.3492, E11.3493, E11.3499, E11.351, E11.3511, E11.3512, E11.3513, E11.3519, E11.3521, E11.3522, E11.3523, E11.3529, E11.3531, E11.3532, E11.3533, E11.3539, E11.3541, E11.3542, E11.3543, E11.3549, E11.3551, E11.3552, E11.3553, E11.3559, E11.359, E11.3591, E11.3592, E11.3593, E11.3599, E11.36, E11.37X1, E11.37X2, E11.37X3, E11.37X9, E11.39, E11.40, E11.41, E11.42, E11.43, E11.44, E11.49, E11.51, E11.52, E11.59, E11.610, E11.618, E11.620, E11.621, E11.622, E11.628, E11.630, E11.638, E11.641, E11.649, E11.65, E11.69, E11.8, E11.9, E13.00, E13.01, E13.10, E13.11, E13.21, E13.22, E13.29, E13.311, E13.319, E13.321, E13.3211, E13.3212, E13.3213, E13.3219, E13.329, E13.3291, E13.3292, E13.3293, E13.3299, E13.331, E13.3311, E13.3312, E13.3313, E13.3319, E13.339, E13.3391, E13.3392, E13.3393, E13.3399, E13.341, E13.3411, E13.3412, E13.3413, E13.3419, E13.349, E13.3491, E13.3492, E13.3493, E13.3499, E13.351, E13.3511, E13.3512, E13.3513, E13.3519, E13.3521, E13.3522, E13.3523, E13.3529, E13.3531, E13.3532, E13.3533, E13.3539, E13.3541, E13.3542, E13.3543, E13.3549, E13.3551, E13.3552, E13.3553, E13.3559, E13.359, E13.3591, E13.3592, E13.3593, E13.3599, E13.36, E13.39, E13.40, E13.41, E13.42, E13.43, E13.44, E13.49, E13.51, E13.52, E13.59, E13.610, E13.618, E13.620, E13.621, E13.622, E13.628, E13.630, E13.638, E13.641, E13.649, E13.65, E13.69, E13.8, E13.9 (any DX on the claim) |
| **Prior Stroke** | G45.0, G45.1, G45.2, G45.8, G45.9, G46.0, G46.1, G46.2, G46.3, G46.4, G46.5, G46.6, G46.7, G46.8, G97.31, G97.32, I60.00, I60.01, I60.02, I60.10, I60.11, I60.12, I60.20, I60.21, I60.22, I60.30, I60.31, I60.32, I60.4, I60.50, I60.51, I60.52, I60.6, I60.7, I60.8, I60.9, I61.0, I61.1, I61.2, I61.3, I61.4, I61.5, I61.6, I61.8, I61.9, I63.00, I63.011, I63.012, I63.013, I63.019, I63.02, I63.031, I63.032, I63.039, I63.09, I63.10, I63.111, I63.112, I63.113, I63.119, I63.12, I63.131, I63.132, I63.133, I63.139, I63.19, I63.20, I63.211, I63.212, I63.213, I63.219, I63.22, I63.231, I63.232, I63.233, I63.239, I63.29, I63.30, I63.311, I63.312, I63.313, I63.319, I63.321, I63.322, I63.323, I63.329, I63.331, I63.332, I63.333, I63.339, I63.341, I63.342, I63.343, I63.349, I63.39, I63.40, I63.411, I63.412, I63.413, I63.419, I63.421, I63.422, I63.423, I63.429, I63.431, I63.432, I63.433, I63.439, I63.441, I63.442, I63.443, I63.449, I63.49, I63.50, I63.511, I63.512, I63.513, I63.519, I63.521, I63.522, I63.523, I63.529, I63.531, I63.532, I63.533, I63.539, I63.541, I63.542, I63.543, I63.549, I63.59, I63.6, I63.8, I63.81, I63.89, I63.9, I66.01, I66.02, I66.03, I66.09, I66.11, I66.12, I66.13, I66.19, I66.21, I66.22, I66.23, I66.29, I66.3, I66.8, I66.9, I67.841, I67.848, I67.89, I97.810, I97.811, I97.820, I97.821 (any DX on the claim)  EXCLUSION: If any of the qualifying claims have any of the following codes in any DX position then EXCLUDE: S01.90XA, S02.0XXA, S02.0XXB, S02.101A, S02.101B, S02.102A, S02.102B, S02.109A, S02.109B, S02.10XA, S02.10XB, S02.110A, S02.110B, S02.111A, S02.111B, S02.112A, S02.112B, S02.113A, S02.113B, S02.118A, S02.118B, S02.119A, S02.119B, S02.11GA, S02.11GB, S02.11HA, S02.11HB, S02.121A, S02.121B, S02.121D, S02.121G, S02.121K, S02.121S, S02.122A, S02.122B, S02.122D, S02.122G, S02.122K, S02.122S, S02.129A, S02.129B, S02.129D, S02.129G, S02.129K, S02.129S, S02.19XA, S02.19XB, S02.2XXA, S02.2XXB, S02.30XA, S02.30XB, S02.31XA, S02.31XB, S02.32XA, S02.32XB, S02.3XXA, S02.3XXB, S02.400A, S02.400B, S02.401A, S02.401B, S02.402A, S02.402B, S02.40AA, S02.40AB, S02.40BA, S02.40BB, S02.40CA, S02.40CB, S02.40DA, S02.40DB, S02.40EA, S02.40EB, S02.40FA, S02.40FB, S02.411A, S02.411B, S02.412A, S02.412B, S02.413A, S02.413B, S02.42XA, S02.42XB, S02.600A, S02.600B, S02.601A, S02.601B, S02.602A, S02.602B, S02.609A, S02.609B, S02.610A, S02.610B, S02.611A, S02.611B, S02.612A, S02.612B, S02.61XA, S02.61XB, S02.620A, S02.620B, S02.621A, S02.621B, S02.622A, S02.622B, S02.62XA, S02.62XB, S02.630A, S02.630B, S02.631A, S02.631B, S02.632A, S02.632B, S02.63XA, S02.63XB, S02.640A, S02.640B, S02.641A, S02.641B, S02.642A, S02.642B, S02.64XA, S02.64XB, S02.650A, S02.650B, S02.651A, S02.651B, S02.652A, S02.652B, S02.65XA, S02.65XB, S02.66XA, S02.66XB, S02.670A, S02.670B, S02.671A, S02.671B, S02.672A, S02.672B, S02.67XA, S02.67XB, S02.69XA, S02.69XB, S02.80XA, S02.80XB, S02.81XA, S02.81XB, S02.82XA, S02.82XB, S02.831A, S02.831B, S02.831D, S02.831G, S02.831K, S02.831S, S02.832A, S02.832B, S02.832D, S02.832G, S02.832K, S02.832S, S02.839A, S02.839B, S02.839D, S02.839G, S02.839K, S02.839S, S02.841A, S02.841B, S02.841D, S02.841G, S02.841K, S02.841S, S02.842A, S02.842B, S02.842D, S02.842G, S02.842K, S02.842S, S02.849A, S02.849B, S02.849D, S02.849G, S02.849K, S02.849S, S02.85XA, S02.85XB, S02.85XD, S02.85XG, S02.85XK, S02.85XS, S02.8XXA, S02.8XXB, S02.91XA, S02.91XB, S02.92XA, S02.92XB, S06.0X0A, S06.0X1A, S06.0X2A, S06.0X3A, S06.0X4A, S06.0X5A, S06.0X6A, S06.0X7A, S06.0X8A, S06.0X9A, S06.1X0A, S06.1X1A, S06.1X2A, S06.1X3A, S06.1X4A, S06.1X5A, S06.1X6A, S06.1X7A, S06.1X8A, S06.1X9A, S06.2X0A, S06.2X1A, S06.2X2A, S06.2X3A, S06.2X4A, S06.2X5A, S06.2X6A, S06.2X7A, S06.2X8A, S06.2X9A, S06.300A, S06.301A, S06.302A, S06.303A, S06.304A, S06.305A, S06.306A, S06.307A, S06.308A, S06.309A, S06.310A, S06.311A, S06.312A, S06.313A, S06.314A, S06.315A, S06.316A, S06.317A, S06.318A, S06.319A, S06.320A, S06.321A, S06.322A, S06.323A, S06.324A, S06.325A, S06.326A, S06.327A, S06.328A, S06.329A, S06.330A, S06.331A, S06.332A, S06.333A, S06.334A, S06.335A, S06.336A, S06.337A, S06.338A, S06.339A, S06.340A, S06.341A, S06.342A, S06.343A, S06.344A, S06.345A, S06.346A, S06.347A, S06.348A, S06.349A, S06.350A, S06.351A, S06.352A, S06.353A, S06.354A, S06.355A, S06.356A, S06.357A, S06.358A, S06.359A, S06.360A, S06.361A, S06.362A, S06.363A, S06.364A, S06.365A, S06.366A, S06.367A, S06.368A, S06.369A, S06.370A, S06.371A, S06.372A, S06.373A, S06.374A, S06.375A, S06.376A, S06.377A, S06.378A, S06.379A, S06.380A, S06.381A, S06.382A, S06.383A, S06.384A, S06.385A, S06.386A, S06.387A, S06.388A, S06.389A, S06.4X0A, S06.4X1A, S06.4X2A, S06.4X3A, S06.4X4A, S06.4X5A, S06.4X6A, S06.4X7A, S06.4X8A, S06.4X9A, S06.5X0A, S06.5X1A, S06.5X2A, S06.5X3A, S06.5X4A, S06.5X5A, S06.5X6A, S06.5X7A, S06.5X8A, S06.5X9A, S06.6X0A, S06.6X1A, S06.6X2A, S06.6X3A, S06.6X4A, S06.6X5A, S06.6X6A, S06.6X7A, S06.6X8A, S06.6X9A, S06.810A, S06.811A, S06.812A, S06.813A, S06.814A, S06.815A, S06.816A, S06.817A, S06.818A, S06.819A, S06.820A, S06.821A, S06.822A, S06.823A, S06.824A, S06.825A, S06.826A, S06.827A, S06.828A, S06.829A, S06.890A, S06.891A, S06.892A, S06.893A, S06.894A, S06.895A, S06.896A, S06.897A, S06.898A, S06.899A, S06.9X0A, S06.9X1A, S06.9X2A, S06.9X3A, S06.9X4A, S06.9X5A, S06.9X6A, S06.9X7A, S06.9X8A, S06.9X9A, OR Z51.89 as the principal DX Code then EXCLUDE |
| **Chronic Obstructive Pulmonary Disease** | J40, J41.0, J41.1, J41.8, J42, J43.0, J43.1, J43.2, J43.8, J43.9, J44.0, J44.1, J44.9, J47.0, J47.1, J47.9 (any DX on the claim) |
| **Chronic Kidney Disease with or without Dialysis** | A18.11, A52.75, B52.0, C64.1, C64.2, C64.9, C68.9, D30.00, D30.01, D30.02, D41.00, D41.01, D41.02, D41.10, D41.11, D41.12, D41.20, D41.21, D41.22, D59.3, E08.21, E08.22, E08.29, E08.65, E09.21, E09.22, E09.29, E10.21, E10.22, E10.29, E10.65, E11.21, E11.22, E11.29, E11.65, E13.21, E13.22, E13.29, E74.8, I12.0, I12.9, I13.0, I13.10, I13.11, I13.2, I70.1, I72.2, K76.7, M10.30, M10.311, M10.312, M10.319, M10.321, M10.322, M10.329, M10.331, M10.332, M10.339, M10.341, M10.342, M10.349, M10.351, M10.352, M10.359, M10.361, M10.362, M10.369, M10.371, M10.372, M10.379, M10.38, M10.39, M32.14, M32.15, M35.04, N00.0, N00.1, N00.2, N00.3, N00.4, N00.5, N00.6, N00.7, N00.8, N00.9, N00.A, N01.0, N01.1, N01.2, N01.3, N01.4, N01.5, N01.6, N01.7, N01.8, N01.9, N01.A, N02.0, N02.1, N02.2, N02.3, N02.4, N02.5, N02.6, N02.7, N02.8, N02.9, N02.A, N03.0, N03.1, N03.2, N03.3, N03.4, N03.5, N03.6, N03.7, N03.8, N03.9, N03.A, N04.0, N04.1, N04.2, N04.3, N04.4, N04.5, N04.6, N04.7, N04.8, N04.9, N04.A, N05.0, N05.1, N05.2, N05.3, N05.4, N05.5, N05.6, N05.7, N05.8, N05.9, N05.A, N06.0, N06.1, N06.2, N06.3, N06.4, N06.5, N06.6, N06.7, N06.8, N06.9, N06.A, N07.0, N07.1, N07.2, N07.3, N07.4, N07.5, N07.6, N07.7, N07.8, N07.9, N07.A, N08, N13.1, N13.2, N13.30, N13.39, N14.0, N14.1, N14.2, N14.3, N14.4, N15.0, N15.8, N15.9, N16, N17.0, N17.1, N17.2, N17.8, N17.9, N18.1, N18.2, N18.3, N18.30, N18.31, N18.32, N18.4, N18.5, N18.6, N18.9, N19, N25.0, N25.1, N25.81, N25.89, N25.9, N26.1, N26.9, Q61.02, Q61.11, Q61.19, Q61.2, Q61.3, Q61.4, Q61.5, Q61.8, Q62.0, Q62.2, Q62.10, Q62.11, Q62.12, Q62.31, Q62.32, Q62.39, R94.4 (any DX on the claim) |
| **Atrial Fibrillation** | I48.0, I48.1, I48.11, I48.19, I48.2, I48.20, I48.21, I48.91 (ONLY first or second DX on the claim) |
| **Ischemic Heart Disease** | I20.0, I20.1, I20.8, I20.9, I21.01, I21.02, I21.09, I21.11, I21.19, I21.21, I21.29, I21.3, I21.4, I21.A1, I21.A9, I22.0, I22.1, I22.2, I22.8, I22.9, I23.0, I23.1, I23.2, I23.3, I23.4, I23.5, I23.6, I23.7, I23.8, I24.0, I24.1, I24.8, I24.9, I25.10, I25.110, I25.111, I25.118, I25.119, I25.2, I25.3, I25.41, I25.42, I25.5, I25.6, I25.700, I25.701, I25.708, I25.709, I25.710, I25.711, I25.718, I25.719, I25.720, I25.721, I25.728, I25.729, I25.730, I25.731, I25.738, I25.739, I25.750, I25.751, I25.758, I25.759, I25.760, I25.761, I25.768, I25.769, I25.790, I25.791, I25.798, I25.799, I25.810, I25.811, I25.812, I25.82, I25.83, I25.84, I25.89, I25.9 (any DX on the claim) |
| **Congestive Heart Failure** | I09.81, I11.0, I13.0, I13.2, I50.1, I50.20, I50.21, I50.22, I50.23, I50.30, I50.31, I50.32, I50.33, I50.40, I50.41, I50.42, I50.43, I50.810, I50.811, I50.812, I50.813, I50.814, I50.82, I50.83, I50.84, I50.89, I50.9 (any DX on the claim) |
| **Home Oxygen** | E0424-E0486 or E1353-E1406 |
| **Alzheimer’s Disease** | F01.50, F01.51, F02.80, F02.81, F03.90, F03.91, F04, F05, F06.1, F06.8, G13.8, G30.0, G30.1, G30.8, G30.9, G31.01, G31.09, G31.1, G31.2, G94, R41.81, R54 (any DX on the claim) |
| **Rheumatoid Arthritis/Osteoarthritis** | M05.00, M05.011, M05.012, M05.019, M05.021, M05.022, M05.029, M05.031, M05.032, M05.039, M05.041, M05.042, M05.049, M05.051, M05.052, M05.059, M05.061, M05.062, M05.069, M05.071, M05.072, M05.079, M05.09, M05.20, M05.211, M05.212, M05.219, M05.221, M05.222, M05.229, M05.231, M05.232, M05.239, M05.241, M05.242, M05.249, M05.251, M05.252, M05.259, M05.261, M05.262, M05.269, M05.271, M05.272, M05.279, M05.29, M05.30, M05.311, M05.312, M05.319, M05.321, M05.322, M05.329, M05.331, M05.332, M05.339, M05.341, M05.342, M05.349, M05.351, M05.352, M05.359, M05.361, M05.362, M05.369, M05.371, M05.372, M05.379, M05.39, M05.40, M05.411, M05.412, M05.419, M05.421, M05.422, M05.429, M05.431, M05.432, M05.439, M05.441, M05.442, M05.449, M05.451, M05.452, M05.459, M05.461, M05.462, M05.469, M05.471, M05.472, M05.479, M05.49, M05.50, M05.511, M05.512, M05.519, M05.521, M05.522, M05.529, M05.531, M05.532, M05.539, M05.541, M05.542, M05.549, M05.551, M05.552, M05.559, M05.561, M05.562, M05.569, M05.571, M05.572, M05.579, M05.59, M05.60, M05.611, M05.612, M05.619, M05.621, M05.622, M05.629, M05.631, M05.632, M05.639, M05.641, M05.642, M05.649, M05.651, M05.652, M05.659, M05.661, M05.662, M05.669, M05.671, M05.672, M05.679, M05.69, M05.70, M05.711, M05.712, M05.719, M05.721, M05.722, M05.729, M05.731, M05.732, M05.739, M05.741, M05.742, M05.749, M05.751, M05.752, M05.759, M05.761, M05.762, M05.769, M05.771, M05.772, M05.779, M05.79, M05.7A, M05.80, M05.811, M05.812, M05.819, M05.821, M05.822, M05.829, M05.831, M05.832, M05.839, M05.841, M05.842, M05.849, M05.851, M05.852, M05.859, M05.861, M05.862, M05.869, M05.871, M05.872, M05.879, M05.89, M05.8A, M05.9, M06.00, M06.011, M06.012, M06.019, M06.021, M06.022, M06.029, M06.031, M06.032, M06.039, M06.041, M06.042, M06.049, M06.051, M06.052, M06.059, M06.061, M06.062, M06.069, M06.071, M06.072, M06.079, M06.08, M06.09, M06.0A, M06.1, M06.20, M06.211, M06.212, M06.219, M06.221, M06.222, M06.229, M06.231, M06.232, M06.239, M06.241, M06.242, M06.249, M06.251, M06.252, M06.259, M06.261, M06.262, M06.269, M06.271, M06.272, M06.279, M06.28, M06.29, M06.30, M06.311, M06.312, M06.319, M06.321, M06.322, M06.329, M06.331, M06.332, M06.339, M06.341, M06.342, M06.349, M06.351, M06.352, M06.359, M06.361, M06.362, M06.369, M06.371, M06.372, M06.379, M06.38, M06.39, M06.80, M06.811, M06.812, M06.819, M06.821, M06.822, M06.829, M06.831, M06.832, M06.839, M06.841, M06.842, M06.849, M06.851, M06.852, M06.859, M06.861, M06.862, M06.869, M06.871, M06.872, M06.879, M06.88, M06.89, M06.8A, M06.9, M08.00, M08.011, M08.012, M08.019, M08.021, M08.022, M08.029, M08.031, M08.032, M08.039, M08.041, M08.042, M08.049, M08.051, M08.052, M08.059, M08.061, M08.062, M08.069, M08.071, M08.072, M08.079, M08.08, M08.09, M08.0A, M08.1, M08.20, M08.211, M08.212, M08.219, M08.221, M08.222, M08.229, M08.231, M08.232, M08.239, M08.241, M08.242, M08.249, M08.251, M08.252, M08.259, M08.261, M08.262, M08.269, M08.271, M08.272, M08.279, M08.28, M08.29, M08.2A, M08.3, M08.40, M08.411, M08.412, M08.419, M08.421, M08.422, M08.429, M08.431, M08.432, M08.439, M08.441, M08.442, M08.449, M08.451, M08.452, M08.459, M08.461, M08.462, M08.469, M08.471, M08.472, M08.479, M08.48, M08.4A, M08.80, M08.811, M08.812, M08.819, M08.821, M08.822, M08.829, M08.831, M08.832, M08.839, M08.841, M08.842, M08.849, M08.851, M08.852, M08.859, M08.861, M08.862, M08.869, M08.871, M08.872, M08.879, M08.88, M08.89, M08.90, M08.911, M08.912, M08.919, M08.921, M08.922, M08.929, M08.931, M08.932, M08.939, M08.941, M08.942, M08.949, M08.951, M08.952, M08.959, M08.961, M08.962, M08.969, M08.971, M08.972, M08.979, M08.98, M08.99, M08.9A, M15.0, M15.1, M15.2, M15.3, M15.4, M15.8, M15.9, M16.0, M16.10, M16.11, M16.12, M16.2, M16.30, M16.31, M16.32, M16.4, M16.50, M16.51, M16.52, M16.6, M16.7, M16.9, M17.0, M17.10, M17.11, M17.12, M17.2, M17.30, M17.31, M17.32, M17.4, M17.5, M17.9, M18.0, M18.10, M18.11, M18.12, M18.2, M18.30, M18.31, M18.32, M18.4, M18.50, M18.51, M18.52, M18.9, M19.011, M19.012, M19.019, M19.021, M19.022, M19.029, M19.031, M19.032, M19.039, M19.041, M19.042, M19.049, M19.071, M19.072, M19.079, M19.09, M19.111, M19.112, M19.119, M19.121, M19.122, M19.129, M19.131, M19.132, M19.139, M19.141, M19.142, M19.149, M19.171, M19.172, M19.179, M19.19, M19.211, M19.212, M19.219, M19.221, M19.222, M19.229, M19.231, M19.232, M19.239, M19.241, M19.242, M19.249, M19.271, M19.272, M19.279, M19.29, M19.90, M19.91, M19.92, M19.93, M45.0, M45.1, M45.2, M45.3, M45.4, M45.5, M45.6, M45.7, M45.8, M45.9, M47.011, M47.012, M47.013, M47.014, M47.015, M47.016, M47.019, M47.021, M47.022, M47.029, M47.10, M47.11, M47.12, M47.13, M47.20, M47.21, M47.22, M47.23, M47.24, M47.25, M47.26, M47.27, M47.28, M47.811, M47.812, M47.813, M47.814, M47.815, M47.816, M47.817, M47.818, M47.819, M47.891, M47.892, M47.893, M47.894, M47.895, M47.896, M47.897, M47.898, M47.899, M47.9, M48.8X1, M48.8X2, M48.8X3, M48.8X4, M48.8X5, M48.8X6, M48.8X7, M48.8X8, M48.8X9 (any DX on the claim) |
| **Depression** | F31.30, F31.31, F31.32, F31.4, F31.5, F31.60, F31.61, F31.62, F31.63, F31.64, F31.75, F31.76, F31.77, F31.78, F31.81, F32.0, F32.1, F32.2, F32.3, F32.4, F32.5, F32.9, F33.0, F33.1, F33.2, F33.3, F33.40, F33.41, F33.42, F33.8, F33.9, F34.1, F43.21, F43.23 (any DX on the claim) |
| **Osteoporosis** | M81.0, M81.6, M81.8 (any DX on the claim) |
| **Cardiogenic Shock** | R57.0 |
| **Prior Percutaneous Coronary Intervention** | Z95.5 |
| **Prior Cardiac Surgery** | Z95.1, Z95.2, Z95.3, Z95.4 |
| **CABG** | 0210093, 0210098, 0210099, 021009C, 021009F, 021009W, 02100A3, 02100A8, 02100A9, 02100AC, 02100AF, 02100AW, 02100J3, 02100J8, 02100J9, 02100JC, 02100JF, 02100JW, 02100K3, 02100K8, 02100K9, 02100KC, 02100KF, 02100KW, 02100Z3, 02100Z8, 02100Z9, 02100ZC, 02100ZF, 0211093, 0211098, 0211099, 021109C, 021109F, 021109W, 02110A3, 02110A8, 02110A9, 02110AC, 02110AF, 02110AW, 02110J3, 02110J8, 02110J9, 02110JC, 02110JF, 02110JW, 02110K3, 02110K8, 02110K9, 02110KC, 02110KF, 021110KW, 02110Z3, 02110Z8, 02110Z9, 02110ZC, 02110ZF, 0212093, 0212098, 0212099, 021209C, 021209F, 021209W, 02120A3, 02120A8, 02120A9, 02120AC, 02120AF, 02120AW, 02120J3, 02120J8, 02120J9, 02120JC, 02120JF, 02120JW, 02120K3, 02120K8, 02120K9, 02120KC, 02120KF, 02120KW, 02120Z3, 02120Z8, 02120Z9, 02120ZC, 02120ZF, 0213093, 0213098, 0213099, 021309C, 021309F, 021309W, 02130A3, 02130A8, 02130A9, 02130AC, 02130AF, 02130AW, 02130J3, 02130J8, 02130J9, 02130JC, 02130JF, 02130JW, 02130K3, 02130K8, 02130K9, 02130KC, 02130KF, 02130KW, 02130Z3, 02130Z8, 02130Z9, 02130ZC, 02130ZF |
| **Valvular surgery other than aortic valve procedure** | 02RG07Z, 02RG08Z, 02RG0JZ, 02RG0KZ, 02RH07Z, 02RH08Z, 02RH0JZ, 02RH0KZ, 02RJ07Z, 02RJ08Z, 02RJ0JZ, 02RJ0KZ, 02QG0ZE, 02QG0ZZ, 02QG3ZE, 02QG3ZZ, 02QG4ZE, 02QG4ZZ, 02QH0ZZ, 02QH3ZZ, 02QH4ZZ, 02QJ0ZG, 02QJ0ZZ, 02QJ3ZG, 02QJ3ZZ, 02JQ4ZG, 02QJ4ZZ |
| **Thoracic Aorta or Aortic Arch Procedure** | 02QX0ZZ, 02QX3ZZ, 02QX4ZZ, 02RX07Z, 02RX0JZ, 02RX0KZ, 02RX47Z, 02RX4JZ, 02RX4KZ, 021X09A, 021X09B, 021X09D, 021X09P, 021X09Q, 021X09R, 021X0AA, 021X0AB, 021X0AD, 021X0AP, 021X0AQ, 021X0AR, 021X0JA, 021X0JB, 021X0JD, 021X0JP, 021X0JQ, 021X0JR, 021X0KA, 021X0KB, 021X0KD, 021X0KD, 021X0KP, 021X0KQ, 021X0KR |
| **Intraoperative or Post-procedural Renal Failure** | N99.0 |
| **Post-procedural Stroke After Cardiac Surgery** | I97.820 |
| **Post-procedure Respiratory Insufficiency Following Thoracic Surgery** | J95.1 |
| **Post-procedural Cardiac Arrest Following Cardiac Surgery** | I97.120 |
| **Insertion of Pacemaker or Defibrillator During Admission** | 0JH604, 0JH605, 0JH606, 0JH607, 0JH608,OJH609 |

CABG: coronary artery bypass graft; SAVR: surgical aortic valve replacement; TAVR: transcatheter aortic valve replacement
