# Supplementary material for: Surgical Aortic Valve Outcomes With Transcatheter Aortic Valve Replacement Hospital Status
Source: Ann Thorac Surg Short Rep. 2025 Jul 30;4(1):87–93. doi: 10.1016/j.atssr.2025.07.002 (PMC13100802; doi:10.1016/j.atssr.2025.07.002)
Supplement: Supplementary Table 2 [file mmc2.docx]

Supplemental Table 2. Mortality Following Isolated SAVR.

|  | **SAVR-only Hospital**  ***(n=423)*** | **SAVR/TAVR Hospital**  ***(n=14,188)*** | **p-value** |
| --- | --- | --- | --- |
| **30-day mortality** | 15 (3.6) | 448 (3.2) | 0.76 |
| **1-year mortality** | 25 (5.9) | 811 (5.7) | 0.99 |

SAVR: surgical aortic valve replacement; TAVR: transcatheter aortic valve replacement
