# Supplementary material for: Surgical Aortic Valve Outcomes With Transcatheter Aortic Valve Replacement Hospital Status
Source: Ann Thorac Surg Short Rep. 2025 Jul 30;4(1):87–93. doi: 10.1016/j.atssr.2025.07.002 (PMC13100802; doi:10.1016/j.atssr.2025.07.002)
Supplement: Supplementary Table 3 [file mmc3.docx]

Supplemental Table 3. Mortality Following SAVR at Hospitals that Performed their First TAVR from 2020-2023.

|  | **SAVR at SAVR/TAVR Hospital Before First TAVR**  ***(n=518)*** | **SAVR at SAVR/TAVR Hospital After First TAVR**  ***(n=437)*** | **p-value** |
| --- | --- | --- | --- |
| **30-day mortality** | 32 (6.2) | 27 (6.2) | >0.99 |
| **1-year mortality** | 60 (11.6) | 33 (7.6) | 0.047 |

SAVR: surgical aortic valve replacement; TAVR: transcatheter aortic valve replacement
