# Supplementary material for: Surgical Aortic Valve Outcomes With Transcatheter Aortic Valve Replacement Hospital Status
Source: Ann Thorac Surg Short Rep. 2025 Jul 30;4(1):87–93. doi: 10.1016/j.atssr.2025.07.002 (PMC13100802; doi:10.1016/j.atssr.2025.07.002)
Supplement: Supplementary Table 4 [file mmc4.docx]

| Supplemental Table 4. Comparison of Post-Operative Morbidity Outcomes of SAVR at SAVR-only Hospitals and SAVR/TAVR Hospitals from 2018-2023. | **SAVR-only Hospital**  ***(n=3,833)*** | **SAVR/TAVR Hospital**  ***(n=94,170)*** | **p-value** |
| --- | --- | --- | --- |
| **Renal Failure** | 24 (0.63) | 798 (0.85) | 0.14 |
| **Respiratory Insufficiency** | 120 (3.1) | 5,222 (5.6) | <0.001 |
| **Stroke** | 19 (0.5) | 612 (0.65) | 0.24 |
| **Cardiac Arrest** | 23 (0.6) | 430 (0.46) | 0.2 |
| **Pacemaker Placement** | 224 (5.8) | 6,771 (7.2) | <0.001 |

SAVR: surgical aortic valve replacement; TAVR: transcatheter aortic valve replacement
