# Supplementary material for: Surgical Aortic Valve Outcomes With Transcatheter Aortic Valve Replacement Hospital Status
Source: Ann Thorac Surg Short Rep. 2025 Jul 30;4(1):87–93. doi: 10.1016/j.atssr.2025.07.002 (PMC13100802; doi:10.1016/j.atssr.2025.07.002)
Supplement: Supplementary Figure Legend [file mmc5.docx]

Supplemental Figure 1. Multivariable Model of 90-day Mortality Following All SAVR (Top) and 90-day Mortality Following Isolated SAVR (Bottom)

SAVR: surgical aortic valve replacement
